# Clinical Characteristics and the Long-Term Post-recovery Manifestations of the COVID-19 Patients—A Prospective Multicenter Cross-Sectional Study

**DOI:** 10.3389/fmed.2021.663670

**Published:** 2021-08-17

**Authors:** Abu Taiub Mohammed Mohiuddin Chowdhury, Md Rezaul Karim, Md. Ahasan Ali, Jahirul Islam, Yarui Li, Shuixiang He

**Affiliations:** ^1^Department of Gastroenterology, First Affiliated Hospital of Xi'an Jiaotong University, Xi'an, China; ^2^Ministry of Health and Family Welfare (OSD-DGHS), Dhaka, Bangladesh; ^3^Hubei Key Laboratory of Embryonic Stem Cell Research, Institute of Neuroscience, Hubei University of Medicine, Shiyan, China; ^4^Department of Pathology and Pathophysiology, Xi'an Jiaotong University, Xi'an, China; ^5^Department of Epidemiology and Health Statistics, Xi'an Jiaotong University, Xi'an, China

**Keywords:** COVID-19, SARS-CoV-2, clinical characteristics, post-recovery manifestations, Bangladesh, post-COVID-19 syndrome

## Abstract

**Objective:** Coronavirus disease 2019 (COVID-19) or severe acute respiratory syndrome coronavirus 2 (SARS-CoV-2) infection is a global issue. In addition to managing acute cases, post-COVID-19 persisting symptoms/complaints and different hematological values are of great concern. These have an impact on the patient's well-being and are yet to be evaluated. Therefore, clinical and primary diagnosis based on routine laboratory findings bears high importance during the initial period of COVID-19, especially in regions with fewer diagnostic facilities.

**Methods:** Clinical information and associated complaints of the COVID-19 illness confirmed by reverse transcription-polymerase chain reaction (RT-PCR) were collected directly from the patients. Regular follow-ups were obtained on the phone every 2 weeks following recovery for 20 weeks. Initial hematological and radiology findings of the hospitalized patients except for intensive care unit (ICU) and high dependency units (HDUs) and a follow-up evaluation after 4 weeks following recovery were analyzed.

**Results:** The post-COVID-19 persisting symptoms/complaints were found among 21.4% of symptomatic patients, which persisted for ≥20 weeks and had a significant relationship with the duration of COVID-19 illness and the existing comorbidity (*p* < 0.05). Post-COVID-19 primary type 2 diabetes mellitus (DM, 0.64%) and hypertension (HTN, 1.28%) and unstable DM (54.55%) and HTN (34.78%) to the pre-existing diabetic and hypertensive patients were observed. Post-recovery remarkable changes in the laboratory values included leukocytosis (16.1%), lymphocytosis (14.5%), and an increased prothrombin time (PT, 25.8%). Abnormalities in the D-dimer, serum ferritin, hemoglobin, and erythrocyte sedimentation rate (ESR) levels were present to an extent. Laboratory findings like chest X-ray, ESR, white blood cell (WBC) count, lymphocyte count, C-reactive protein (CRP), serum glutamic pyruvic transaminase (SGPT), serum ferritin, PT, D-dimer, and serum creatinine are important markers for the diagnosis and prognosis of COVID-19 illness (*p* < 0.05).

**Conclusion:** Post-COVID-19 persisting symptoms and the changes in the laboratory values need to be considered with importance and as a routine clinical measure. Post-COVID-19 periodic follow-up for evaluating the patient's physical condition and the biochemical values should be scheduled with care and managed accordingly to prevent future comorbidity in patients with the post-COVID-19 syndrome.

## Introduction

Coronavirus disease 2019 (COVID-19) or severe acute respiratory syndrome coronavirus 2 (SARS-CoV-2) infection is a current global pandemic caused by an RNA virus of the beta Coronavirus family (1). After the first report in the Wuhan province of China, the disease quickly spread all over the globe. However, the presentation of COVID-19 varies depending on the region of origin. Thus, in a region with inadequate facilities, primary identification of SARS-CoV-2 infection remains a challenge. Furthermore, the overlapping clinical presentation of COVID-19 with local viral flu often misleads the diagnosis, thus causing morbidity, mortality, and further spread of the infection.

Additionally, like an emerging disease, managing the persisting symptoms/complaints and the abnormality of the laboratory values following the post-COVID-19 recovery period is complex. Proper understanding of these complaints is necessary for both the healthcare professionals and the patients, as it has a broad impact on patient well-being and health status. The changes in the laboratory values following COVID-19 recovery are yet to be evaluated. This manuscript is intended to investigate the acute and post-recovery manifestations of COVID-19 illness, including the post-COVID-19 persisting symptoms/complaints and changes in the laboratory parameter, namely, post-COVID-19 syndrome among the patients in Bangladesh.

## Methods

### Study Population and Data Collection

The list of SARS-CoV-2 confirmed cases by reverse transcription-polymerase chain reaction (RT-PCR) was identified and collected from the local health facilities of the Chattogram division in Bangladesh. All the patients took treatments in different COVID-19 designated hospitals. Preliminary information of the symptomatic cases that required treatments regarding the COVID-19 illnesses was collected directly from the outpatient department (OPD) and the hospitalized patients. These included disease symptoms, history, comorbid condition, and associated complaints. In addition, initial radiology and laboratory tests such as chest computed tomography (CT), X-ray chest P/A view, hemoglobin level, erythrocyte sedimentation rate (ESR), total and differential counts of white blood cell (WBC), red blood cell (RBC), platelet, C-reactive protein (CRP), serum glutamic pyruvic transaminase (SGPT), serum ferritin, prothrombin time (PT), D-dimer, and serum creatinine were performed, and the values were noted. In addition, febrile patients were tested for dengue NS1 antigen, dengue immunoglobulin G (IgG) and immunoglobulin M (IgM) antibody, Salmonella Typhi/Para-Typhi IgM and IgG antibody, immunochromatographic test (ICT) for malaria, Widal test to exclude dengue, malaria, and enteric fever.

### Ethical Consideration and Consent

Ethical committee approval was taken from Xi'an Jiaotong University, China. Informed written consent was taken in every case. In the case of the below 16 years old participants, written informed consent was obtained from a parent or guardian.

### Exclusion Criteria

Critically ill COVID-19 patients in the intensive care unit (ICU) and high dependency units (HDUs); patients who had a preexisting uncontrolled comorbid condition with compromised organ function such as severe chronic obstructive pulmonary disease (COPD) exacerbation, advanced ischemic heart disease, severe uncontrolled diabetes mellitus (DM), advanced renal and hepatic disease, and advanced stage of carcinoma; and prehospitalized (not due to COVID-19), immunocompromised, and pregnant patients were not included in this study. In addition, those who had a recent history of hematological, biochemical, or chest radiograph abnormality within the last 30 days were excluded. Expired patients during this study who missed the regular follow-up schedule twice and who missed the follow-up laboratory evaluation and those who were unwilling to continue participation were also excluded from the study.

### Post-COVID-19 Persisting Symptoms and Hematological Manifestations

COVID-19 recovery was defined by two negative PCR 7 days apart. The recovery time was calculated from the date of the first negative PCR. COVID-19 symptoms or newer symptoms, which persisted or appeared following the COVID-19 recovery, were identified as persisting symptoms. Regular follow-ups were obtained on the phone every 2 weeks from each participant to identify and analyze the post-COVID-19 persisting symptoms/complaints. This process continued for 20 weeks. The symptoms that may have been attributed to other illnesses during this study by any current or chronic condition were carefully examined and not included in the study.

An initial laboratory assessment while hospitalized and a post-COVID-19 follow-up evaluation after 4 weeks were done to understand the changes. The follow-up laboratory evaluations included X-ray chest P/A view, a complete blood count (hemoglobin level, ESR, total and differential count of WBC, RBC, platelet), CRP, SGPT, serum ferritin, PT, D-dimer, and serum creatinine. In addition, patients with persisting fever were tested for dengue, malaria, and enteric fever. In the cases of laboratory abnormalities, treatments were given to the patients; therefore, no further laboratory follow-up data were collected.

### Data and Statistical Analysis

Data were presented as mean ± standard deviation, and statistical analysis was carried out using SPSS V25.0. Column statistics, chi-square test, and a *t*-test with a 95% confidence interval were done to see the significance of the necessary values. *p* < 0.5 was considered statistically significant.

## Results

### Demographics and Mortality Rate Among the Study Population

Initially, 512 symptomatic COVID-19 cases were included. Among them, 53 patients refused to participate in the study. Out of the rest, 83 were hospitalized, and 376 were OPD cases. While continuing treatment, 12 OPD patients required hospitalization. Later, they were included among the hospitalized patients. Therefore, 95 hospitalized and 364 OPD cases were enrolled. After enrollment, 33 hospitalized patients were excluded during the study; 11 had preexisting comorbid conditions that fit the exclusion criteria, 10 died during the treatment, and 12 were lost during follow-up. Among the OPD participants, 113 were excluded from the study; 45 patients had preexisting conditions, 48 were unable to maintain regular follow-up, and 20 were unwilling to continue participating. Finally, 313 patients were included in the study for analysis, among whom 62 were hospitalized and 251 were OPD patients. The mortality rate among the COVID-19 patients in our study was 1.96%. The hospitalization rate among the OPD-treated patients was 3.2%.

### General Characteristics of the Study Population

The total number of patients was 313; 251 (80.2%) male and 62 (19.8%) female. The mean age was 37.74 years, range from 3 to 75 years. Patients according to the age-groups were 3 (1–10 years), 19 (11–20 years), 91 (21–30 years), 82 (31–40 years), 56 (41–50 years), 40 (51–60 years), and 22 (60+ years). The age-group 21–30 years was the most affected, consisting of 91 (29.1%) patients. Among the hospitalized patients (*n* = 62), (19.80%), 55 (88%) were male and 7 (11.29%) were female. In addition, 239 (74%) patients experienced <10 days and 74 (23.6%) experienced >10 days of COVID-19-related symptoms. Sixty-seven (21.4%) of the total study population had post-COVID-19 persisting symptoms/complaints. Moreover, 20.4% of the patients had comorbidities such as hypertension (HTN) [21 (6.7%)] and Type 2 diabetes mellitus (T2DM) [20 (6.4%)] (Table 1).

**Table 1:** General characteristics of the study population.

**Variables**		**Number of cases (*n*)**	**Percentage %**
Number of patients (*n* = 313)	M	251	80.2
	FM	62	19.8
Age (in years)	Minimum	3 years	
	Maximum	75 years	
	Mean ± SD	37.74 ± 13.70	
	Median	35	
	1–10 years	3	1.0
	11–20 years	19	6.1
	21–30 years	91	29.1
	31–40 years	82	26.2
	41–50 years	56	17.9
	51–60 years	40	12.8
	60+ years	22	7
Hospitalization (*n* = 62, 19.80%)	M	55	88
	FM	7	11.29
	0–10 days	34 (M 29, FM 5)	54.8
	>10 days	28 (M 26, FM 2)	45.2
Duration of COVID-19 symptoms	0–10 days	239 (M 185, FM 54)	76.4
	>10 days	74 (Male 66, FM 8)	23.6
Recovery duration (confirmation of case to negative PCR)	1–10 days	157 (M 120, FM 37)	50.2
	11–20 days	133 (M 112, FM 21)	42.5
	>21 days	23 (M 19, FM 4)	7.3
Post-COVID-19 persisting symptoms/complaints	None	246 (M 198, FM 48)	78.6
	Present	67 (M 53, FM 14)	21.4
Comorbidity 64 (20.4%) None 249 (79.6%)	Cancer	1	0.3
	Gastritis	1	0.3
	Hypotension	1	0.3
	Bronchial asthma	13	4.2
	T2DM	20	6.4
	HTN	21	6.7
	Ischemic heart disease	2	0.6
	Liver disease	1	0.3
	Skin Allergy	1	0.3
	HBs Ag (+Ve)	1	0.3
	HTN and T2DM	2	0.6

*COVID-19, coronavirus disease 2019; FM, female; HTN, hypertension; M, male; T2DM, Type 2 diabetes mellitus*.

### Clinical Characteristics of COVID-19 Patients

Most COVID-19 patients presented with fever [274 (87.5%)] and cough [103 (32.9%)]. Other symptoms were myalgia [54 (17.25%)], loss of appetite [51 (16.30%)], headache [43 (13.74%)], abdominal cramps/pain [34 (11.50%)], difficulty in breathing [19 (6.07%)], loss of smell [15 (5.11%)], fatigue [16 (5.11%)], chest tightness [11 (3.11%)], vomiting [9 (2.86%)], non-specific discomfort–unable to describe [9 (2.86%)], rhinorrhea [9 (2.86%)], sore throat [7 (2.24%)], insomnia [6 (1.91%)], diarrhea [5 (1.60%)], sneezing [5 (1.60%)], burning sensation in the body [4 (1.28%)], partial loss of hearing [2 (0.64%)], neck pain [1 (0.32%)], joint pain [1 (0.32%)], chest pain [1 (0.32%)], and itching [1 (0.32%)]. Nonetheless, anxiety was experienced by all of these patients at a certain level (Table 2).

**Table 2:** COVID-19 symptoms among the study population.

**Name of the symptoms**	**Number of patients (*n*)**	**Percentage %**
Fever	274	87.5
Cough	103	32.9
Myalgia	54	17.25
Loss of appetite	51	16.30
Headache	43	13.74
Abdominal cramp/pain	36	11.50
Difficulty in breathing	19	6.07
Loss of smell	16	5.11
Fatigue	16	5.11
Chest tightness	11	3.51
Vomiting	9	2.86
Non-specific discomfort	9	2.86
Rhinorrhea	9	2.86
Sore throat	7	2.24
Insomnia	6	1.91
Diarrhea	5	1.60
Sneezing	5	1.60
Burning sensation in the body	4	1.28
Partial loss of hearing	2	0.64
Loss of taste	1	0.32
Neck pain	1	0.32
Joint pain	1	0.32
Chest pain	1	0.32
Itching	1	0.32

*COVID-19, coronavirus disease 2019*.

A significant difference was found in a *t*-test among initial radiological and hematological parameters with the post-COVID-19 recovery follow-ups. These were chest X-ray consolidated, ESR, the total count of WBC, lymphocyte, CRP, serum ferritin, PT, D-dimer, and serum creatinine levels (*p* < 0.05). However, no significant differences were found in the neutrophil, RBC, platelet, and hemoglobin levels (*p* > 0.05; Table 3).

**Table 3:** Comparison between the initial on admission laboratory and radiology finding and post-COVID-19 recovery (following negative PCR) 4-week follow-up.

**Parameters**	**Initial findings during admission**	**Post-COVID-19 4-week follow-up**	***t*-test (95% CI)**
Chest X-ray consolidation	13.36 ± 11.35	0	*p* < 0.0001****
Hemoglobin	13.28 ± 1.55	13.35 ± 1.75	*p* = 0.824
ESR	31.42 ± 19.41	18.89 ± 9.83	*p* < 0.0001****
WBC total count	7,162 ± 3,005	1,035 ± 1,999	*p* = 0.036**
RBC	4.94 ± 6.78	5.16 ± 0.70	*p* = 0.0837
Platelet	230,859 ± 66,213	232,016 ± 75,655	*p* = 0.93
Neutrophil	65.97 ± 12.71	62.75 ± 8.21	*p* = 0.0741
Lymphocyte	28.42 ± 12.52	36.24 ± 9.56	*p* < 0.0001****
Monocyte	3.78 ± 2.34	3.33 ± 2.32	*p* = 0.289
Eosinophil	1.75 ± 1.24	2.08 ± 1.16	*p* = 0.57
Basophil	0.067 ± 0.25	0.080 ± 0.27	*p* = 0.77
CRP	29.86 ± 23.65	5.3 ± 9.07	*p* < 0.0001****
SGPT	62.71 ± 45.18	49.13 ± 23.35	*p* = 0.0385*
Serum ferritin	543.3 ± 460.6	162 ± 105.2	*p* < 0.0001****
Prothrombin time	11.67 ± 1.92	12.59 ± 1.74	*p* = 0.0063**
D-dimer	1.05 ± 1.24	0.08 ± 0.18	*p* < 0.0001****
Serum creatinine	1.028 ± 0.27	0.85 ± 0.26	*p* = 0.0003***

*Data are presented as mean ± SD. For the reference values, please see Table 7*.

COVID-19, coronavirus disease 2019; CRP, C-reactive protein; ESR, erythrocyte sedimentation rate; RBC, red blood cell; SGPT, serum glutamic pyruvic transaminase; WBC, white blood cell. Level of significance of the p-value *, **, ***, ****.

### Post-COVID-19 Persisting Symptoms/Complaints and Their Characteristics

Out of the total, 78.6% (246) patients had no complaints following recovery. The post-COVID-19 persisting symptoms were lethargy [23 (7.4%)], cough [16 (5.1%)], chest discomfort and pain [12 (3.8%)], breathlessness on activity [12 (3.8%)], fatigue [12 (3.8%)], significant anxiety with lack of concentration and occasional amnesia that impaired daily activity [5 (1.6%)], headache [4 (1.2%)], low-grade fever [2 (0.6%)], and joint pain [2 (0.6%)]; mild body ache/chill/back pain was experienced by one (0.3%) each. Following recovery from COVID-19, 2 (0.64%) patients developed T2DM and 4 (1.28%) developed HTN. Nine (55.55%) of the total diabetic patients faced difficulties controlling the glycemic levels and had to take additional hypoglycemic therapy. Eight (34.78%) of the total hypertensive patients had to increase the dose or take additional antihypertensive therapy to achieve proper blood pressure control (Table 4).

**Table 4:** Post-COVID-19 persisting symptoms/complaints and comorbidity.

**Name of the persisting symptom**	**Number of patients (*n*)**	**Percentage of the patients %**
No complaint	246	78.6
Lethargy	23	7.4
Cough	16	5.1
Chest discomfort and pain	12	3.8
Fatigue	12	3.8
Breathlessness on activity	12	3.8
Anxiety, lack of concentration, and occasional amnesia	5	1.6
Headache	4	1.2
Fever (persisting fever)	2	0.6
Joint pain	2	0.6
Mild body ache	1	0.3
Chill	1	0.3
Enteric fever	1	0.3
Back pain	1	0.3
T2DM (following COVID-19)	2	0.64
HTN (following COVID-19)	4	1.28
Post-COVID uncontrolled DM	9	54.55
Post-COVID uncontrolled HTN	8	34.78

*COVID-19, coronavirus disease 2019; HTN, hypertension; T2DM, Type 2 diabetes mellitus*.

Furthermore, 0.6% of participants had the earliest relief from post-COVID-19 persisting symptoms on the third week from the recovery date. Fifteen (4.8%) recovered on the 10th week. Seven (2.2%) patients experienced 20 weeks or more duration of the persisting complaints (Table 5). However, the correlations between the duration of COVID-19 symptoms of 0–10 days and >10 days and post-COVID-19 persisting symptoms of 0–8 weeks and >8 weeks were statistically significant (^**^p = 0.00; Table 6).

**Table 5:** Duration of the post-COVID-19 complaints or symptoms.

**Duration (in weeks)**	**Number of patients**	**Percentage of the patients %**
None	246	78.6
3rd	2	0.6
4th	3	1.0
5th	9	2.9
6th	3	1.0
7th	11	3.5
8th	6	1.9
9th	2	0.6
10th	15	4.8
15th	5	1.6
16th	4	1.3
20 or more	7	2.2
Total	313	100.0

**Table 6:** Relation between the duration of the COVID-19 symptoms and the duration of post-COVID-19 persisting complaints.

**Duration of the COVID-19 symptoms**	**Duration of the post-COVID-19 complaints**			**Number of cases (*n*)**	
	**0–8 weeks**	**>8 weeks**	**None**		
0–10 days	21	31	187	239	
>10 days	13	02	59	74	Chi-square (*X^2^*) *p* = 0.00^**^
Total	34	33	246	313	

*COVID-19, coronavirus disease 2019*.

** *Level of significance*.

### Initial and Post-recovery Laboratory Manifestations of COVID-19 Cases

The mean age was 43.32 years (21–75 years). Forty-three (69.35%) patients had consolidations in the chest X-ray during admission, while at 4-week follow-up, three (0.48%) patients had mild features of pneumonitis. Lymphocytopenia was detected among 19 (30.6%). CRP was increased in all the patients during admission but in 11 (17.7%) during the follow-up. ESR, WBC, neutrophil, serum ferritin, D-dimer, and PT were increased in 43 (69.4%), 8 (12.9%), 11 (17.7%), 28 (45.1%), 31 (50%), and 14 (22.6%) patients during admission, while during follow-up, they were 34 (54.8%), 10 (16.1%), 4 (33.8%), 6 (9.7%), 4 (6.4%), and 16 (25.8%) subsequently. Males had a higher percentage (52.7%) of increased D-dimer during admission. Erythrocytopenia was found only in females. Moreover, females had a greater presentation of an increase in the CRP level (42.8%), serum ferritin (14.2%), PT (42.8%), lymphocyte (28.6%), and X-ray (mild features of pneumonitis) (14.2%) during the follow-up. However, D-dimer (7.3%) and total WBC (16.3%) levels were increased in the males (Table 7).

**Table 7:** Primary (on admission) hematological findings and the post-COVID-19 recovery (following negative PCR) 4-week follow-up of the hospitalized patients.

**Parameters**	**Initial findings (admission day)**			**Post-COVID-19 4-week follow-up**		
	**Patients (*n*)**	**Male**	**Female**	**Patients**	**Male**	**Female**
Chest X ray	62	55	7	3 (0.48%)	2 (3.6%)	1 (14.2%)
Decreased Hb	31 (50%)	28 (50.9%)	4 (57.1%)	26 (41.9%)	23 (41.8%)	3 (42.8%)
Increased ESR	43 (69.4%)	39 (70.9%)	4 (57.1%)	34 (54.8%)	30 (54.5%)	4 (57.1%)
Leukocytosis	8 (12.9%)	6 (10.9%)	2 (28.6%)	10 (16.1%)	9 (16.3%)	1 (14.2%)
Decreased RBC	4 (6.5%)	0	4 (57.1%)	3 (4.84%)	0	3 (42.8%)
Platelet	0	0	0	0	0	0
Neutrophil	↑11 (17.7%)	↑10 (18.2%)	↑1 (14.3%)	↑4 (33.8%)	↑4 (7.2%)	0
Lymphocyte	**↓**11 (17.7%)	**↓**10 (18.2%)	**↓**1 (14.3%)	↑9 (14.5%)	↑7 (12.7%)	↑2 (28.6%)
Monocyte ↑↓	0	0	0	0	0	0
Esinophil ↑↓	0	0	0	0	0	0
Basophil ↑↓	0	0	0	0	0	0
Increased CRP	62 (100%)	55 (100%)	7 (100%)	11 (17.7%)	8 (14.5%)	3 (42.8%)
Increased SGPT	18 (29%)	16 (29%)	2 (28.6%)	3 (4.8%)	3 (5.4%)	0
Increased ferritin	28 (45.1%)	25 (45.5%)	3 (42.9%)	6 (9.7%)	5 (9.1%)	1 (14.2%)
Increased PT	14 (22.6%)	11 (20%)	3 (42.9%)	16 (25.8%)	13 (23.6%)	3 (42.8%)
Increased D-dimer	31 (50%)	29 (52.7%)	2 (28.6%)	4 (6.4%)	4 (7.3%)	0
Increased creatinine	2 (3.2%)	2 (3.6%)	0	0	0	0

*The total number of patients (n = 62); Male: 55, Female: 7. Reference value: Hb (g/dl): Female: 12–16; Male: 14–18; ESR (mm in first hour): Female: 0–15; Male: 0–10; WBC/CC: 4,000–11,000; RBC (Million/CC): 4.0–6.2; Platelet/CC: 150,000–450,000; Neutrophil: 40%−75%; Lymphocyte: 20–45%; Monocyte: 2–10%; Eosinophil: 1–6%; Basophil: 0–1%; CRP (mg/dl): <3; SGPT (Units/L) Male: 16–63 Female: 14–59; Serum ferritin (ng/ml): Male: 13–370, Female: 9–253; PT (s): 0–13. COVID-19, coronavirus disease 2019; CRP, C-reactive protein; ESR, erythrocyte sedimentation rate; Hb, hemoglobin; PT, prothrombin time; RBC, red blood cell; SGPT, serum glutamic pyruvic transaminase; WBC, white blood cell. ↑increase value, ↓decrease value*.

### Subgroup Analysis of the Persisting Symptoms of COVID-19 Cases

Lethargy mainly was seen in age-group 21–30 years (nine patients) and cough (four patients each) in age-groups 21–30, 31–40, 41–50, 51–60 years. Chest pain and breathlessness on activity were dominant in 31–40 years (six patients) and anxiety in 11–20 years (four patients). Four patients 51–60 years of age complained of headache, and two patients each among those aged 41–50 and 31–40 years complained of breathlessness on activity and joint pain. Back pain and myalgia were experienced by two patients of the age-group 21–30 years. In addition, 53 (11.2%) males and 14 (4.5%) females had post-COVID-19 persisting symptoms. Chest pain (12), breathlessness on activity (2), and joint pain (2) were solely complained about by the male participants. On the other hand, six female participants suffered from persisting fever with headache and lethargy. The difference between patients having persisting symptoms according to gender was not statistically significant (*p* = 0.277) (Supplementary Table 1).

According to age-group, subgroup analyses of patients with persisting symptoms and the duration of the persisting symptoms were done. This revealed that 1–10 years had no complaints, and the 41–50 years age-group had post-COVID-19 complaints that persisted for >20 weeks. The age-groups 21–30 and 41–50 years started to recover the earliest at 3 weeks and the complain persisted up to 16 weeks, and 8 weeks post-recovery respectively; and those were 41–50 years complained up to 20+ weeks (Supplementary Table 2).

### Subgroup Analysis of Duration of the Persisting Symptoms Among the Comorbid Patients

A total of 26 patients who experienced post-COVID-19 persisting symptoms had comorbidity. HTN was found in 11 patients; among these patients, four patients experienced a delayed recovery from the post-COVID-19 symptoms/complaints about ≥20 weeks. T2DM and bronchial asthma were found among 12 patients (six each). They had an earlier recovery from the post-COVID-19 persisting symptoms on the 15th and 12th week. Among the persisting symptoms, fever was experienced by hypertensive patients. Lethargy was complained about by 12 patients; among these 12 patients, five had HTN, four had T2DM, two had bronchial asthma, and one had skin allergy. Persisting cough was complained about by five patients, and four who had bronchial asthma suffered from chest pain and breathlessness on activity until the 10th week following a negative PCR. A significant relationship was found between the comorbidity and the duration of the post-COVID-19 persisting symptoms *p* = 0.000 in the chi-square test (Supplementary Table 3).

## Discussion

SARS-CoV-2 is a systemic infection causing a cytotoxic storm that exerts an effect on the homeostatic mechanism (2). The incubation period of COVID-19 can be up to 14 days. Presenting symptoms of COVID-19 may differ from mild to a severe illness expressed by respiratory distress, chest tightness, or pain. COVID-19 symptoms might present with fever, cough, myalgia, chest tightness, breathing difficulty, sore throat, and loss of taste or smell. A deteriorating sign of SARS-CoV-2 infection includes central cyanosis, confusion, respiratory difficulty, persistent chest pain, heaviness in the chest, and inability to stay awake (3). Studies have reported differences in presenting symptoms of COVID-19 illness, depending on regions. However, several symptoms are common among all the reports. Hematological manifestations of COVID-19 are also different depending on the geographical locations and socioeconomic conditions. Moreover, post-COVID-19 persisting symptoms/complaints and associated laboratory manifestations are yet to be evaluated.

The impact of the patient's clinical characteristics and post-recovery manifestations of the COVID-19 patients will shed light on this disease's systematic management (4). Initial investigations from China have shown that clinical symptoms such as fever, cough, dyspnea, and hematological findings of COVID-19 patients are like those in SARS-CoV (5–8). Heterogeneous clinical manifestations have been discovered in COVID-19 patients from different areas of China (9–16), Europe (17, 18), US (19, 20), Malaysia (21), Jordan (22), Kuwait (23), South Korea (24), and Africa (25). Thus, understanding the clinical features can help clinicians predict the disease's progression and apply possible intervention approaches.

This study evaluated the clinical characteristics of COVID-19 illness, post-COVID-19 persisting symptoms/complaints, and changes in the laboratory findings following COVID-19 recovery among the patients in Bangladesh. Our study contains 313 symptomatic patients 3–75 years of age. Among them, 251 (80.2%) were male and 62 (19.8%) were female. The age-groups 20–30 years and 31–40 years were the most affected by SARS-CoV-2 infection, 91 (29.1%) and 82 (26.2%). Overall, 50.2% of the patients recovered within 10 days, 42.5% recovered within 10–20 days, and 7.3% required >21 days to recover. Sixty-seven (21.4%) of the total participants experienced varying degrees of persisting symptoms/complaints following COVID-19 recovery that persisted up to 20 weeks and more. Fifty-three (21.11%) of the males and 14 (22.5%) of the female participants had experienced post-COVID-19 persisting complaints. The distribution of these symptoms was almost similar in both genders. Furthermore, 21.6% of the COVID-19 patients had comorbid conditions, e.g., T2DM, HTN, ischemic heart disease, and bronchial asthma. Diabetic and hypertensive patients were 6.4 and 6.7% (Table 1).

Moreover, in our study, the duration of COVID-19 symptoms and the duration of the post-COVID-19 persisting symptoms/complaints had a significant correlation (^****^p = 0.00; Table 6). This signifies a longer duration of the illness caused by the prolongation of the post-recovery symptoms.

According to recent studies, COVID-19 symptoms can occasionally continue for months. Thus, the SARS-CoV-2 virus can increase the incidence of long-standing health complications. Carfi et al. (26), in their study, found that in patients who had recovered from COVID-19, 87.4% reported persistence of at least one symptom, especially fatigue and dyspnea. Balachandar et al. (27) also conducted a longitudinal study to evaluate the COVID-19 recovered patient's health status, which revealed that COVID-19 might have the potential impact to cause multiorgan damage. Another study on post-COVID-19 recovery in patients with or without preexisting diabetes by Akter et al. (28) found that people with diabetes have faced severe COVID-19 manifestation and post disease problems. Greenhalgh et al. (29) also reported that nearly 10% of individuals experience persistent illness after COVID-19 infection. Subsequently, another study also reported persisting symptoms of COVID-19 (30). However, studies on persisting symptoms of COVID-19 are scanty. Therefore, it is suggested that follow-up study of COVID-19 recovered patients would be useful to assess any changes in the other organs.

According to this study, COVID-19 patients in this region presented with fever, cough, myalgia, loss of appetite, headache, abdominal cramps, breathing difficulty, loss of smell, fatigue, chest tightness, vomiting, rhinorrhea, sore throat, insomnia, diarrhea, and sneezing (Table 2). However, all participants complained about anxiety, though it was not reported as a symptom in this study. A total of 78% of the participants had no complaints following recovery. Though all the persisting symptoms found in our study caused impairment of routine activity, 2 (0.64%) had developed T2DM and 4 (1.28%) patients developed HTN following recovery. Many preexisting diabetic and hypertensive patients experienced difficulties controlling their normal level and had to receive additional therapy.

The extent of the post-COVID-19 persisting symptoms was at least 3 weeks up to 20 weeks and more. The greatest number of recoveries from post-COVID-19 persisting complaints was on the 10th week, followed by the seventh and fifth weeks among the participants (Table 5). However, the very early age-group of 0–10 years had no report of persisting symptoms (Supplementary Tables 1, 2). According to the initial hematological findings (Table 7), CRP was increased in all the patients. A notable percentage of patients had an increased ESR, WBC, CRP, SGPT, serum ferritin, PT, D-dimer, and serum creatinine level. Approximately 50% of patients had decreased hemoglobin levels, and 6.5% had decreased RBC levels.

An increased percentage of lymphocytosis, leukocytosis, and high PT was observed in the 4-week post-recovery follow-up (Tables 3, 7). No patient had consolidation on chest X-ray during post-COVID-19 4-week follow-up, except three patients who had mild pneumonitis features. However, the new increase in WBC level and PT is a matter of deep concern. There were significant differences in several of these parameters between initial findings during admission and post-COVID-19 4-week follow-up. These parameters are chest X-ray consolidated, ESR, WBC, lymphocyte, CRP, SGPT, serum ferritin, PT, D-dimer, and serum creatinine level (*p* < 0.05; Table 3). This proves the reliability and high accuracy of these parameters in the diagnosis and prognosis of COVID-19 illness. Evaluation of the underlying cause and management of increased PT, lymphocyte, and WBC demand close attention in the case of post-COVID-19 patients. Though it might be a positive feedback response to hemodilution therapy, further study is required for a better understanding of the cause and management of this condition.

Our study had several limitations. The sample size was relatively small; the follow-up radiology and laboratory values were evaluated only once after 4 weeks; critically ill patients in the ICU were not included. Recovery analysis based on the different drug treatments was not done, and the death cases were not accessed. Despite the limitations, we tried to analyze the clinical features and post-recovery manifestations of the COVID-19 cases without any existing comorbid conditions.

## Conclusion

COVID-19 epidemic is a global concern. Primary diagnosis of SARS-CoV2 infection is crucial for the proper management and control of the epidemic, especially in the low facility regions where a PCR and CT chest are not readily available. In our study, 21.4% of the symptomatic COVID-19 patients suffered from persisting symptoms/complaints following recovery, which might persist for more than 20 weeks. They included lethargy, cough, chest discomfort and pain, breathlessness on activity, fatigue, anxiety with lack of concentration and occasional amnesia, headache, persistent low-grade fever, joint pain, body ache, chill, and back pain. Post-COVID-19 development of T2DM (0.64%) and HTN (1.28%) and also the development of unstable DM (54.55%) and HTN (34.78%) to the pre-existing DM and hypertensive patients are a matter of immediate concern. The post-COVID-19 significant changes in the laboratory values included leukocytosis (16.1%), lymphocytosis (14.5%), and an increased PT (25.8%). Besides, an abnormality in the D-dimer, serum ferritin, hemoglobin, and ESR levels was also present to an extent. Moreover, existing comorbidity among the COVID-19 patients increases the risk and duration of the post-COVID-19 persisting symptoms. Management of the post-COVID-19 persisting symptoms and the abnormal laboratory values appeared to be a challenge for healthcare professionals. Better understanding of these conditions is equally important for the patients. A periodic follow-up and evaluation of the patient's physical condition and the biochemical values will help prevent future comorbidity in patients with the post-COVID-19 syndrome.

## Data Availability Statement

The original contributions presented in the study are included in the article/Supplementary Material, further inquiries can be directed to the corresponding author/s.

## Ethics Statement

Ethical Committee approval was taken from Xi'an Jiaotong University, China. Written informed consent to participate in this study was provided by the participants' legal guardian/next of kin.

## Author Contributions

AM: research concept, data collection, data interpretation, statistical analysis, manuscript writing, and editing. MK: data collection, manuscript writing, and editing. MA: statistical analysis. JI: data collection. YL: interpreted the data of COVID-19 patients. SH: review of the manuscript, maintained supervision, and ensured the quality of the research. All authors contributed to the article and approved the submitted version.

## Conflict of Interest

The authors declare that the research was conducted in the absence of any commercial or financial relationships that could be construed as a potential conflict of interest.

## Publisher's Note

All claims expressed in this article are solely those of the authors and do not necessarily represent those of their affiliated organizations, or those of the publisher, the editors and the reviewers. Any product that may be evaluated in this article, or claim that may be made by its manufacturer, is not guaranteed or endorsed by the publisher.

## Abbreviations

COVID-19: coronavirus disease 2019
CDC: Centers for Disease Control and Prevention
CRP: C-reactive protein
CT: computed tomography
DM: diabetes mellitus
ESR: erythrocyte sedimentation rate
HTN: hypertension
IgG: immunoglobulin G
IgM: immunoglobulin M
IEDCR: Institute of Epidemiology, Disease Control and Research
LMWH: low-molecular weight heparin
MERS: Middle East respiratory syndrome
OPD: outpatient department
PT: prothrombin time
RBC: red blood cell
RT-PCR: reverse transcription-polymerase chain reaction
SARS-CoV-2: severe acute respiratory syndrome coronavirus 2
SGPT: serum glutamic pyruvic transaminase
T2DM: Type 2 diabetes mellitus
WBC: white blood cell.
