# Supplementary material for: Clinical Characteristics and the Long-Term Post-recovery Manifestations of the COVID-19 Patients—A Prospective Multicenter Cross-Sectional Study
Source: Front Med (Lausanne). 2021 Aug 17;8:663670. doi: 10.3389/fmed.2021.663670 (PMC8416537; doi:10.3389/fmed.2021.663670)
Supplement: Supplementary file 1 [file Table_1.docx]

**Supplementary Table S1**: Sub-group analysis of the Persisting symptoms according to the age group and gender.

| Persisting Symptoms | Age group (In years) | | | | | | | Number  (n) | According to the gender | | |
| --- | --- | --- | --- | --- | --- | --- | --- | --- | --- | --- | --- |
|  | 1-10 | 11-20 | 21-30 | 31-40 | 41-50 | 51-60 | 60+ |  | Male | Female | Chi-square test |
| None | 3 | 14 | 70 | 68 | 42 | 29 | 20 | 246 | 198 | 48 | [Male and female]  P= 0.277 |
| Joint pain | 0 | 0 | 0 | 2 | 0 | 0 | 0 | 2 | 2 | 0 |  |
| breathlessness on activity | 0 | 0 | 0 | 0 | 2 | 0 | 0 | 2 | 2 | 0 |  |
| Fever, Headache | 0 | 0 | 0 | 0 | 0 | 4 | 0 | 4 | 0 | 4 |  |
| Enteric fever | 0 | 0 | 1 | 0 | 0 | 0 | 0 | 1 | 1 | 0 |  |
| Anxiety, restless | 0 | 4 | 0 | 0 | 1 | 0 | 0 | 5 | 1 | 4 |  |
| Back pain | 0 | 0 | 1 | 0 | 0 | 0 | 0 | 1 | 1 | 0 |  |
| Chest pain, breathlessness on activity | 0 | 0 | 3 | 6 | 3 | 0 | 0 | 12 | 12 | 0 |  |
| Cough | 0 | 1 | 4 | 4 | 4 | 1 | 2 | 16 | 14 | 2 |  |
| Fever, lethargy | 0 | 0 | 2 | 0 | 0 | 0 | 0 | 2 | 0 | 2 |  |
| Lethargy | 0 | 0 | 9 | 2 | 4 | 6 | 0 | 21 | 19 | 2 |  |
| Myalgia | 0 | 0 | 1 | 0 | 0 | 0 | 0 | 1 | 1 | 0 |  |
| Total (n) | 3 | 19 | 91 | 82 | 56 | 40 | 22 | 313 | 251 | 62 |  |
