# Supplementary material for: Clinical Characteristics and the Long-Term Post-recovery Manifestations of the COVID-19 Patients—A Prospective Multicenter Cross-Sectional Study
Source: Front Med (Lausanne). 2021 Aug 17;8:663670. doi: 10.3389/fmed.2021.663670 (PMC8416537; doi:10.3389/fmed.2021.663670)
Supplement: Supplementary file 2 [file Table_2.docx]

**Supplementary Table S2**: Subgroup analysis of patients with persisting symptoms according

to age group and the duration of the persisting symptoms (in weeks).

| Age in years | Duration of Post-COVID19 complain (Weeks) | | | | | | | | | | | Total  Patient (n) |
| --- | --- | --- | --- | --- | --- | --- | --- | --- | --- | --- | --- | --- |
|  | 3 | 4 | 5 | 6 | 7 | 8 | 9 | 10 | 15 | 16 | 20+ |  |
| 0-10 | 0 | 0 | 0 | 0 | 0 | 0 | 0 | 0 | 0 | 0 | 0 | 0 |
| 11-20 | 0 | 0 | 0 | 0 | 0 | 0 | 0 | 4 | 1 | 0 | 0 | 5 |
| 21-30 | 1 | 1 | 0 | 1 | 1 | 4 | 2 | 7 | 0 | 4 | 0 | 21 |
| 31-40 | 0 | 0 | 7 | 0 | 4 | 0 | 0 | 3 | 0 | 0 | 0 | 14 |
| 41-50 | 1 | 2 | 0 | 0 | 2 | 2 | 0 | 0 | 0 | 0 | 7 | 14 |
| 51-60 | 0 | 0 | 0 | 2 | 4 | 0 | 0 | 1 | 4 | 0 | 0 | 11 |
| 60+ | 0 | 0 | 2 | 0 | 0 | 0 | 0 | 0 | 0 | 0 | 0 | 2 |
| (n) | 2 | 3 | 9 | 3 | 11 | 6 | 2 | 15 | 5 | 4 | 7 | 67 |
