# Supplementary material for: Clinical Characteristics and the Long-Term Post-recovery Manifestations of the COVID-19 Patients—A Prospective Multicenter Cross-Sectional Study
Source: Front Med (Lausanne). 2021 Aug 17;8:663670. doi: 10.3389/fmed.2021.663670 (PMC8416537; doi:10.3389/fmed.2021.663670)
Supplement: Supplementary file 3 [file Table_3.docx]

**Supplementary Table S3:** Subgroup analysis of duration of the persisting symptoms (in weeks) among the comorbid patients.

| Comorbidity | Duration of Post COVID19 complain (Weeks) | | | | | | | Total  (n=26) | Pearson  Chi-square (*ᵡ*^2^)  P= 0.00 | Persisting  fever | Back pain  headache | Chest pain, breathlessness on activity | Cough | Lathery |
| --- | --- | --- | --- | --- | --- | --- | --- | --- | --- | --- | --- | --- | --- | --- |
|  | 5 | 6 | 7 | 8 | 10 | 15 | 20+ |  |  |  |  |  |  |  |
| Hypertension | 2 | 0 | 4 | 0 | 1 | 0 | 4 | 11 |  | 4 | 0 | 0 | 2 | 5 |
| T2 DM | 0 | 0 | 0 | 0 | 1 | 5 | 0 | 6 |  | 0 | 0 | 0 | 2 | 4 |
| Bronchial asthma | 0 | 2 | 0 | 0 | 4 | 0 | 0 | 6 |  | 0 | 0 | 4 | 0 | 2 |
| Carcinoma | 0 | 0 | 0 | 1 | 0 | 0 | 0 | 1 |  | 0 | 0 | 0 | 1 | 0 |
| Skin allergy | 1 | 0 | 0 | 0 | 0 | 0 | 0 | 1 |  | 0 | 0 | 0 | 0 | 1 |
| Hepatitis | 1 | 0 | 0 | 0 | 0 | 0 | 0 | 1 |  | 0 | 1 | 0 | 0 | 0 |
| Total | 4 | 2 | 4 | 1 | 6 | 5 | 4 | 26 |  | 4 | 1 | 4 | 5 | 12 |
